# Systematic review of population‐based bladder cancer registries: How criteria heterogeneity affects the comparison of incidences

**DOI:** 10.1002/cam4.5494

**Published:** 2022-12-18

**Authors:** José María Caballero, José María Gili, Juan Camilo Pereira, Alba Gomáriz, Carlos Castillo, Montserrat Martín‐Baranera

**Affiliations:** ^1^ Department of Urology Hospital Universitari Mútua de Terrassa Terrassa Barcelona Spain; ^2^ Department of Pediatrics Obstetrics & Gynecology and Preventative Medicine at the Autonomous University of Barcelona Facultad de Medicina ‐ Edificio M, Campus Universitario UAB Barcelona Spain; ^3^ Department of Clinical Epidemiology Consorci Sanitari Integral Barcelona Spain

**Keywords:** bladder cancer, cancer, epidemiology, incidence, population‐based registry

## Abstract

**Background:**

The population‐based registry of bladder cancer (BC) raises specific problems intrinsic to the tumor, as the inclusion of noninfiltrating, potentially malignant and multiple tumors. We performed a systematic review (PRISMA guidelines) of population‐based BC registries to obtain information on their geographic areas involved, last dates of real incidence of BC, and rules coding used in BC for uncertain behavior, in situ and multiple tumors.

**Methods:**

Using MEDLINE and Google Scholar, we identified scientific publications of in the last 10 years in English or Spanish, whether they were related to a national or international cancer registry, provided information on registry rules, and provided data on the incidence of BC.

**Results:**

After the first screening, a total of 194 references were obtained. After a second analysis, three registries were selected: International Agency for Research on Cancer (IARC) is a world registry providing real incidence of BC in the period 2008–2012. Surveillance, Epidemiology, and End Results (SEER) Program registered incidence until 2017 in more than 90% of the US population. Spanish Network of Cancer Registries (REDECAN) unifies 14 Spanish registries (27.4% of the population) with real incidence data from 2010 to 2015. The coding and inclusion rules have been modified, but currently, most registries include BC in situ and uncertain behavior tumors. Whenever a new case occurs 36 months after a previous diagnosis, SEER registers those as multiple incident cancers in the same location, while IARC and REDECAN only allow one cancer per location during the lifespan of the patient.

**Conclusions:**

Comparison of the incidence of BC among different population‐based cancer registries is prone to bias due to the methodological differences regarding the inclusion of carcinomas in situ, indeterminate, and multiple tumors. A good cancer registry could provide better surveillance strategies for BC patients.

## Graphical abstract

The bladder cancer registry is complex due to the peculiarities of urothelial tumors. The heterogeneity in the rules to define carcinomas in situ, indeterminate and multiple tumors interfere with the comparability of incidence of bladder cancer.

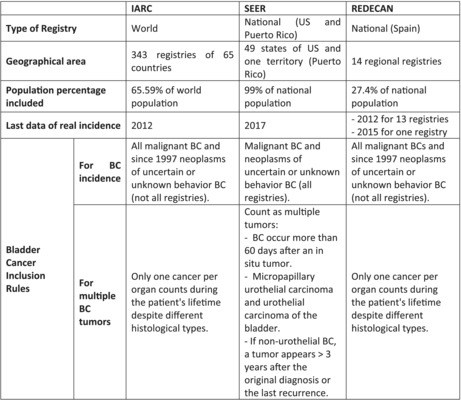

## 1 INTRODUCTION

Population‐based cancer registries are essential to study the incidence of the different types of malignant neoplasms. For these registries to be useful, it is necessary to define the rules for collecting and storing data, to establish which cases are to be recorded, how they are to be coded, and what type of registries are generated.^1^, ^2^, ^3^, ^4^, ^5^ The rules of each registry to define primary tumors or multiple tumors^6^ are an attempt to provide incidence data that are consistent and reproducible.

Bladder cancer (BC) is the most common of those that affect the urinary tract, the majority of them being urothelial. Depending on their pathology, urothelial tumors can be flat lesions (dysplasia, carcinoma in situ), noninvasive papillary neoplasms limited to the mucosa (papilloma, inverted papilloma, papillary neoplasm of low malignant potential, low‐ or high‐grade papillary carcinoma), and invasive papillary neoplasms involving the submucosa or beyond (low‐grade or high‐grade papillary carcinoma). Compared to other types of cancer, the BC registry poses specific problems related above all to the inclusion and coding of noninvasive papillary tumors, carcinoma in situ or indeterminate ones. In addition, BC frequently presents as multiple tumors, and with a high probability of recurrence.^7^, ^8^

There is a wide disparity among BC incidences derived from the coding rules applied, whether the figures represent real or estimated incidences, whether the crude or adjusted rates are considered, and the areas included in the different registries. For this reason, the aim of this paper is to analyze the different methods used to calculate incidence in population‐based BC registries and thus assess their comparability.

## 2 METHODS

A systematic review of the literature on population‐based BC registries and incidence data has been carried out, following the Preferred Reporting Items for Systematic Reviews and Meta‐Analyses (PRISMA)^9^ guide. Identifying scientific publications related to BC registries was the chosen method to locate these registries. Two electronic databases were used, MEDLINE (via Pubmed) and Google Scholar, limited to articles published in the last 10 years in English or Spanish. The search was carried using the following MeSH terms: “urinary bladder neoplasms” and its synonyms, “registries” and “incidence”. We declare that this revision has not been registered.

### 2.1 Registries selection

Two reviewers reviewed all documents separately following the eligibility criteria.

All documents and reports were included, if they met all the inclusion criteria:

1. The documents were related to a national or international cancer registry.
2. The documents and reports provided data about BC incidence.
3. The documents and reports provided data of the cancer registry program or have a website to consult this information.

The exclusion criteria:

1. Studies published prior to January 1, 2011.
2. The documents o reports in which neither a national or international population‐based cancer registry nor a hospital registry were identified.
3. Studies in which clear data on the incidence of BC in a defined period were not identified.
4. Documents in national or international registries in which it has not been possible to analyze the BC incidence registration rules due to incomplete information, not having a website or the information being in a language other than English or Spanish.
5. Documents duplicates.

First, the articles were selected based on their titles and abstracts, according to the previously established inclusion and exclusion criteria. If there were doubts, a second analysis was performed: the full text of the documents was read, subsequently reaching a consensus between the two reviewers on their inclusion or not in the systematic review. The selected articles were grouped according to the registry they were reporting data from. Cancer registry websites were reviewed if necessary. After the second screening, an international registry that covered the greater number of countries and a national registry with the highest number of publications were chosen for further analysis. A third cancer registry was chosen that, in addition to meeting all the inclusion criteria, would provide data on our health area.

### 2.2 Data collection process

Data were extracted from the full text of the articles and from the web pages of each cancer registry. Information was obtained on:

1. The geographic areas involved in each registry, and the last dates of real and estimated incidence of BC.
2. The BC coding rules, with special interest in registration practices related to tumors of uncertain or unknown behavior, carcinoma in situ, multiple tumors, and recurrences.

## 3 RESULTS

### 3.1 Registries selection

**FIGURE 1:**
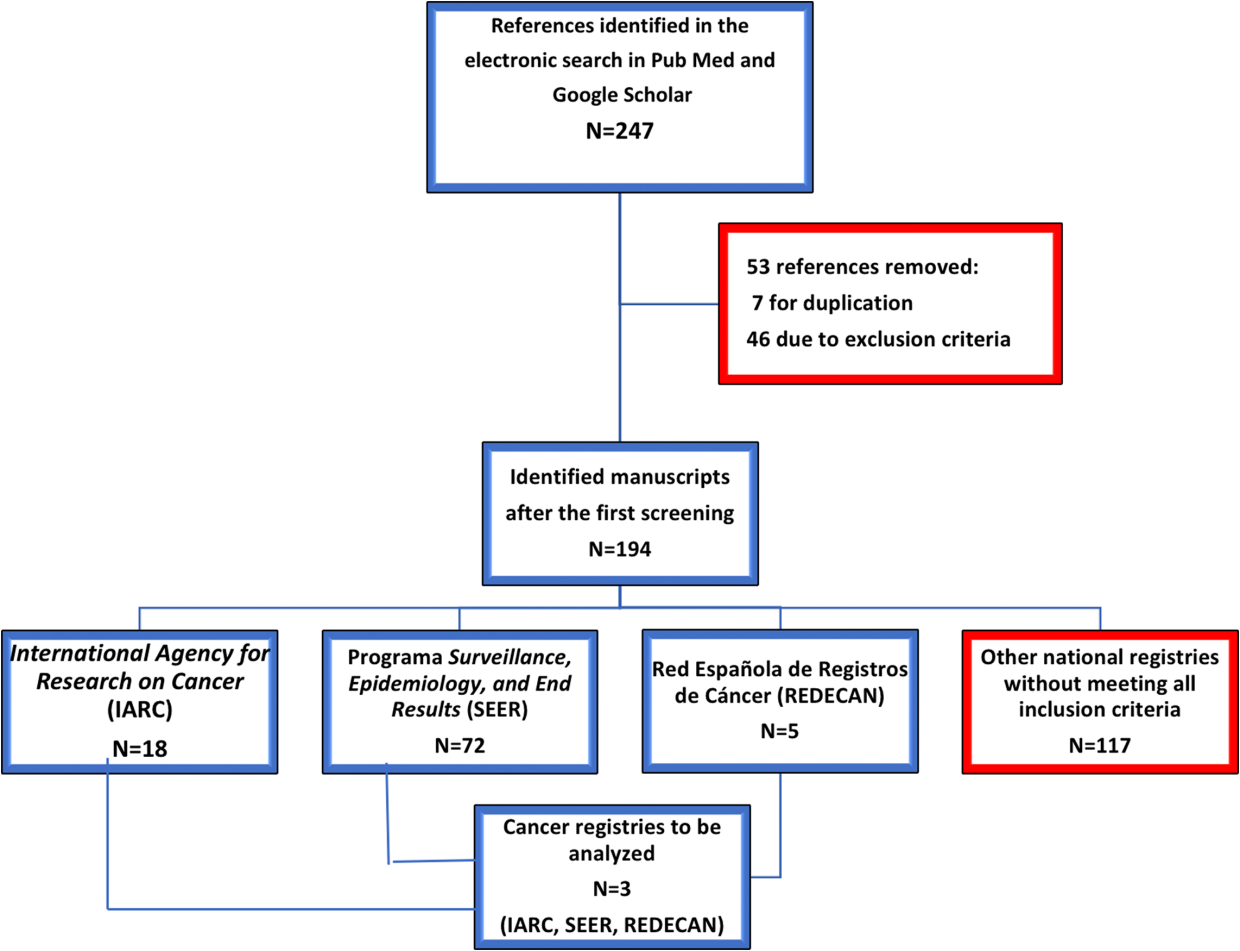
Bladder cancer registry selection flowchart.

Title and abstracts of a total of 247 references were screened based on inclusion and exclusion criteria. After the first screening, a total of 194 references were obtained (Figure 1):

1. 18 articles corresponded to a single international registry: the International Agency for Research on Cancer (IARC) and its two publications, Cancer Incidence in Five Continents (CI5) and GLOBOCAN. Its official website is https://www.iarc.who.int/
2. 72 articles obtained data from one U.S. national registry: Surveillance, Epidemiology, and End Results (SEER) Program of the National Cancer Institute (NCI) that provides information on cancer statistics in the US population (https://seer.cancer.gov/).
3. 5 articles corresponded to data from the national registry of our health area: Spanish Network of Cancer Registries (REDECAN) based on population‐based cancer registries from different provinces of Spain (https://redecan.org).
4. The remaining 117 articles corresponded to national registries from China (21 articles), Denmark (10), France (10), Iran (9), Canada (6), Sweden (8), Japan (8), Italy (6), the United Kingdom (4), South Korea (4), Norway (3), India (3), Australia (3), Germany (3), Austria (2), Morocco (2), Egypt (2) and Cyprus, Lebanon, Ireland, Serbia, the Netherlands, Estonia, Brazil, Mexico, Nigeria, Argentina, Mozambique, Algeria, Jordan with one article per country.

After the second analysis, according to eligibility criteria, an international registry that covered the greater number of countries, a national registry with the highest number of publications and a national registry of our health data were chosen for further analysis: IARC, SEER, and REDECAN.

### 3.2 Data on each registry

The similarities and differences between the three BC registries included in the analysis, in terms of geographic areas and population included, and rules for including BC cases and multiple tumors, are summarized in Table 1.

**TABLE 1:** The similarities and differences between the data on each registry for bladder cancer

		IARC	SEER	REDECAN
Type of registry		World	National (US and Puerto Rico)	National (Spain)
Geographical area		343 registries of 65 countries	49 states of United States and one territory (Puerto Rico)	14 regional registries
Population percentage included		65.59% of world population	99% of national population	27.4% of national population
Last data of real incidence		2012	2017	‐ 2012 for 13 registries ‐ 2015 for one registry
Bladder Cancer Inclusion Rules	For BC incidence	All malignant BC and since 1997 neoplasms of uncertain or unknown behavior BC (not all registries).	Malignant BC and neoplasms of uncertain or unknown behavior BC (all registries).	All malignant BCs and since 1997 neoplasms of uncertain or unknown behavior BC (not all registries).
	For multiple BC tumors	Only one cancer per organ counts during the patient's lifetime despite different histological types.	Count as multiple tumors: ‐ BC occur more than 60 days after an in situ tumor. ‐ Micropapillary urothelial carcinoma and urothelial carcinoma of the bladder. ‐ If non‐urothelial BC, a tumor appears >3 years after the original diagnosis or the last recurrence.	Only one cancer per organ counts during the patient's lifetime despite different histological types.

Abbreviations: BC, Bladder cancer; IARC, International Agency for Research on Cancer; REDECAN, Spanish Network of Cancer Registries; SEER, Surveillance, Epidemiology, and End Results Program (of the National Cancer Institute); US, United States.

#### 3.2.1 International Agency for Research on Cancer (IARC)

##### 3.2.1.1 Geographical areas and dates of cancer registries

The IARC had two publications:

1. The Cancer Incidence in Five Continents (CI5) series publishes every 5 years, detailed information on the incidence of cancer based on registries from around the world. To date, it comprises 11 volumes, beginning in 1960 with the publication of volume I (32 records from 29 countries), and ending with volume XI (343 records from 65 countries).^10^ In total, this world registry covers 465 million inhabitants. Although in some countries the records include practically the entire population, in others they only represent a small percentage. Volume XI was published online in 2020, and reported cancers diagnosed between 2008 and 2012, although it is not necessary to have data for the 5 years included in the period (3 years is sufficient).
2. Since 2001, GLOBOCAN provides estimates of incidence on the different types of cancer by age and sex, with CI5 being the main source of information. The most recent publication, GLOBOCAN 2020,^11^ offers incidence estimates for the year 2020 in 185 countries or territories for 36 types of cancer that include only malignant neoplasms, except BC, which may include carcinoma in situ, or tumors of uncertain or unknown behavior, in incidence (but not mortality), depending on the definitions of malignancy in each cancer registry.

##### 3.2.1.2 Urinary bladder cancer coding rules

In volumes VIII,^12^ IX,^13^ and X^14^ of CI5, the International Classification of Diseases. tenth edition (ICD‐10) (WHO, 1992)^15^ was used. Instead, in volume XI, they used the ICD‐10 version 2010^16^ and the 2011 revision of the International Classification of Diseases for Oncology, third edition (ICD‐O‐3) (WHO, 2013).^17^ Under the term “neoplasia,” ICD‐10 contains a five‐column table with the following headings for each topography: malignant, secondary or metastatic, in situ, benign, and uncertain and unknown behavior. In contrast, ICD‐O‐3 uses only one set of four digits for topography, which remains the same as in ICD‐10 (e.g., C67 bladder), adding another digit depending on the exact location e.g., C67 0.2 is a tumor located on the lateral wall of the bladder). In addition, morphological data is added in the form of four digits that describe the cell type or histology of the tumor, a fifth digit after the slash (/) that describes the behavior (benign /0; uncertain /1; malignant, but not invasive), or in situ/2, malignant invasive/3, metastatic/6); and a sixth digit on the grade, differentiation or phenotype (from grade I if well differentiated to grade IV if very undifferentiated or anaplastic) (Table 2).

**TABLE 2:** Bladder cancer coding according to the International Classification of Diseases for Oncology, third edition (ICD‐O‐3)^16^

TOPOGRAPHY C67 BLADDER C67.0 Trigone of bladder C67.1 Dome of bladder C67.2 Lateral wall of bladder C67.3 Anterior wall of bladder C67.4 Posterior wall of bladder C67.5 Bladder neck Internal urethral orifice C67.6 Ureteric orifice C67.7 Urachus C67.8 Overlapping lesion of bladder C67.9 Bladder. NOS Bladder wall. NOS Urinary bladder. NOS
MORPHOLOGY 4 digits cellular type (histology) 5th digit behavior code for neoplasms /0 Benign /1 Uncertain whether benign or malignant (Borderline malignancy; low malignant potential; uncertain malignant potential) /2 Carcinoma in situ (intraepithelial; no infiltrating; noninvasive) /3 Malignant. primary site /6* Malignant. metastatic site (malignant. secondary site) /9* Malignant. uncertain whether primary or metastatic site * Not used by cancer registries 6th digit code for histological grading and differentiation 1 Grade I Well differentiated (differentiated. NOS) 2 Grade II Moderately differentiated (moderately well differentiated; intermediate differentiation) 3 Grade III Poorly differentiated 4 Grade IV Undifferentiated (anaplastic) 9 Grade or differentiation not determined. not stated or not applicable
812–813 Transitional cell papillomas and carcinomas. Particular cases. 8120/1 Urothelial papilloma. NOS (transitional cell papilloma. NOS; papilloma of bladder (C67._)) 8130/1 Papillary transitional cell neoplasm of low malignant potential (C67._)(papillary urothelial neoplasm of low malignant potential (C67._)) 8120/2 Transitional cell carcinoma in situ (Urothelial carcinoma in situ) 8130/2 Papillary transitional cell carcinoma, non‐invasive (C67._)(Papillary urothelial carcinoma. noninvasive (C67._)) 8120/3 Transitional cell carcinoma. NOS (urothelial carcinoma. NOS; transitional carcinoma) 8130/3 Papillary transitional cell carcinoma (C67._)(papillary urothelial carcinoma (C67._)) 8131/3 Transitional cell carcinoma, micropapillary (C67._)

Currently, most cancer registries collect data on tumors coded based on their behavior as /2 and /3. However, in the case of BC, the situation is different regarding benign tumors, in situ and of uncertain behavior. In volume VI of CI5,^18^ in order to maintain geographic comparability, only cases with a /3 code were included, and thus tumors with benign behavior, in situ, and unspecified were left out. However, when volume VII^19^ was elaborated and the different registries were asked about the codes they used for noninvasive and unspecified diagnoses of malignant BC, many of them reported that they assigned behavior code / 3 to on‐site diagnoses and unspecified, which made it impossible to distinguish these cases. For this reason, the editors of volume VII decided to accept that noninvasive diagnoses of BC were considered malignant by pathologists. Thus, since volume VII, the BC registry has included the categories in situ and unspecified. Subsequently, to edit volume XI of IC5, the different registries were asked again if they included some nonmalignant diagnoses and how they specifically coded bladder carcinoma in situ and unspecified, among others. In this way, in the tables of volume XI, whenever possible, neoplasms of uncertain or unknown behavior are included together with invasive cancers and are indicated by a dagger symbol next to the BC code C67, and an accompanying explanatory note. Some registries preferred not to include such cases in their dataset, even when they were available in the registry, in order to maintain continuity over time that ensured comparability.

Concerning the presence of multiple tumors, according to IARC rules, only the first malignant neoplasm in an organ or group of organs counts. From the rules of the registry of multiple neoplasms of the IARC,^6^ the following highlights are worth noting:

1. The recognition of the existence of two or more primary cancers is not time dependent.
2. A primary cancer is one that originates from a primary site or tissue and is not an extension, recurrence, or metastasis.
3. Multifocal tumors, that is, masses apparently not in continuity with other primary cancers arising from the same site or primary tissue, for example, the bladder, are counted as a single cancer.

##### 3.2.1.3 Incidence of bladder cancer

The latest real incidence data from all the global registries that are part of IARC date from the period 2008 to 2012 were published in CI5 volume XI.^10^

#### 3.2.2 Surveillance, Epidemiology, and End Results (SEER) program

##### 3.2.2.1 Geographic areas and dates of cancer registries

The SEER reports cancer incidence in the US since 1973. Last published in July 2021, incidence data for all cancers from 2001 through 2017 are included.^20^ Data from 49 states and one territory (Puerto Rico) met data criteria for every year during 2013 to 2017, representing 99% of the population of the US and Puerto Rico.^20^

In the SEER program, timely and accurate calculation of cancer incidence rates is slightly hampered by delayed reporting: on November 1, cases diagnosed approximately 2 years earlier are submitted to SEER registries for incidence, exactly 22 months after the end of the specific diagnostic year. These data are published in the spring of the following year. For example, in November 2020, the cases diagnosed through 2018 are submitted and incidence data are published in April 2021.

##### 3.2.2.2 Urinary bladder cancer coding rules

The anatomical location and the histology are coded with the ICD‐O‐3 (Table 2).^17^ Only cases defined as malignant are included, except for BC in which, when reporting incidence, carcinomas in situ and malignant ones are combined.^21^ Multiple tumors are considered and are recorded separately whenever^22^:

1. an invasive tumor occurs more than 60 days after an in situ tumor.
2. the patient has micropapillary urothelial carcinoma and urothelial carcinoma of the bladder.
3. a tumor appears in a patient who has being clinically free of disease for more than 3 years after the original diagnosis or the last recurrence. However, this rule does not apply when both/all tumors are bladder urothelial carcinoma.

##### 3.2.2.3 Incidence of urinary bladder cancer

SEER contributes to BC incidence data in IARC CI5 publications following IARC criteria. Volume X of CI5 (2003–2007)^14^ does not include neoplasms of uncertain or unknown behavior together with invasive cancer, but volume XI (2008–2012)^10^ does. In both volumes, the incidences vary depending on the year being considered, on the number of registries included, and on the consideration of crude or adjusted rate (15.0–30.8/100,000 inhabitants in males) (Table 3). Alternatively, in the SEER publications themselves, which always include neoplasms of uncertain behavior, in situ or unknown, the incidence rates per 100.000 inhabitants are adjusted by age to the US 2000 US standard population of 9, 17, or 21 registries, which also modifies the figures (34.2–37.18/100,000 inhabitants in males) (Table 3).^23^

**TABLE 3:** Comparison of the incidences of bladder cancer in Spain and the USA according to IARC (published in Cancer Incidence in Five Continents vol. X^13^ and XI^9^) and according to web data or publications from REDECAN^23^, ^24^, ^25^, ^26^, ^27^, ^28^, ^29^ and SEER^19,22^

Registry	Publication	Code included ICD 10	Period of years	Men		Women	
				CR	ASR world	CR	ASR world
Albacete	CI5 vol. X CI5 vol. XI	C67 # C67 #	2003–2007 2008–2010	54.5 51.9	28.9 27.7	9.1 8.9	3.8 4.3
	REDECAN	C67. D09	2003–2007		28.0		3.0
Asturias	CI5 vol. X CI5 vol. XI	C67 # C67 #	2003–2007 2008–2010	66.1 75.1	28.8 33.6	13.5 14.4	4.9 4.9
	REDECAN	C67. D09	2003–2007		28.00		4.00
Canarias	CI5 vol. X CI5 vol. XI	C67 # C67 #	2003–2006 2008–2011	40.1 45.1	27.1 27.4	7.3 9.5	4.0 4.6
	REDECAN		2003–2007 2008–2012	28.59 31.14	9.09 18.38	4.66 6.10	2.3 2.62
Castellón (Valencian Community)	CI5 vol. X CI5 vol. XI	C67 #	2008–2012	60.7	34	10.6	4.9
	REDECAN	C67. D09	2013 2004–2012	73.07	36.7 34.00	13.76	4.00
Ciudad Real	CI5 vol. X CI5 vol. XI	C67 C67 #	2004–2007 2008–2011	64.2 60.1	32.1 31.3	9.3 12.1	3.5 5.5
	REDECAN	C67. D09	2003–2007		32.10		3.00
Cuenca	CI5 vol. X CI5 vol. XI	C67 C67 #	2003–2007 2008–2011	56.3 56.2	24.4 25.2	7.6 10.3	2.6 2.9
	REDECAN		2003–2007		24.00		2.00
Euskadi	CI5 vol. X CI5 vol. XI	C67 # C67 #	2003–2007 2008–2012	66.4 74.2	34.1 35.1	12.3 17.0	4.9 6.8
	REDECAN	C65‐C68	2003–2007 2011–2015	65.3 65.3	34.00	16.0 16.0	8.8
Girona	CI5 vol. X CI5 vol. XI	C67 #	2003–2007 2008–2012	66.7 65.7	37.5 36.6	12.2 11.8	5.0 5.1
	REDECAN		2007–2009 2010–2012	68.6	35.00 37.4	12.7	3.00 5.63
Granada	CI5 vol. X CI5 vol. XI	C67 C67 #	2003–2007 2008–2012	54.3 58.0	32.2 32.8	8.1 10.0	4.0 4.5
	REDECAN		2003–2007		32.00		4.00
La Rioja	CI5 vol. X CI5 vol. XI	C67 # C67 #	2003–2007 2008–2012	64.0 70.5	31.6 34.5	12.1 15.2	4.9 5.8
	REDECAN		2003–2007		31.00		4.00
Mallorca	CI5 vol. X CI5 vol. XI	C67 # C67 #	2003–2007 2008–2011	70.0 65.5	44.5 40.4	11.2 10.7	5.3 5.4
	REDECAN		2006–2008		28.6		3.00
Murcia	CI5 vol. X CI5 vol. XI	C67 # C67 #	2003–2007 2008–2010	56.3 62.9	37.9 41.1	9.3 10.4	4.8 5.2
	REDECAN		2003–2007	56.3	37.00	9.3	4.00
Navarra	CI5 vol. X CI5 vol. XI	C67 # C67 #	2003–2007 2008–2010	72.4 77.8	39.8 43.4	14.4 18.1	6.8 8.5
	REDECAN		2003–2007		39.00		6.00
Tarragona	CI5 vol. X CI5 vol. XI	C67 C67 #	2003–2007 2008–2012	66.5 75.4	36.9 40.7	11.1 12.5	5.0 4.9
	REDECAN		2008–2009 2011–2013 2012–2014	71.3 76.4	39.0 36.1 37.7	10.8 12.5	4.0 4.4 5.3
SEER	CI5 vol. X CI5 vol. XI	C67 C67 #	2003–2007 (9 registries) 2003–2007 (18 registries) 2008–2012 (9 registries) 2008–2012 (18 registries)	30.8 29.5 15.8 15.0	21.0 20.8 19.6 19.0	10.0 9.5 5.0 4.7	5.4 5.3 5.1 4.9
	SEER*	C67 #	2003–2007 (9 registries) 2003–2007 (17 registries) 2008–2012 (21 registries) 2014–2018 (21 registries)		37.18 37.22 37.80 34.2		9.26 9.21 9.34 8.5

*Note*: # (including neoplasms of uncertain or unknown behavior together with invasive cancer). ASR world = world age‐standardized incidence rate per 100,000 inhabitants.

Abbreviation: CR, Crude incidence rate per 100,000 inhabitants.

^a^ Rate per 100,000 population adjusted for age to the US 2000 US standard population (19 age groups ‐ census P25‐1130).

#### 3.2.3 Spanish Network of Cancer Registries (REDECAN)

##### 3.2.3.1 Geographic areas and dates of cancer registries

In Spain, REDECAN was established in 2010 based on the population‐based cancer registries of Albacete, Asturias, the Canary Islands, Castellón, Cuenca, Ciudad Real, Girona, Granada, La Rioja, Mallorca, Murcia, Navarra, Euskadi, and Tarragona, which represented 27.4% of the total Spanish population in 2020.

REDECAN also provides BC incidence data to IARC, with the latest published data being from 2012.^10^ Based on other publications of REDECAN or of each of the member registries, and through the European Cancer Information System (ECIS) page (publication of the European Network of Cancer Registries, ENCR),^24^ the real incidence of BC dates from 2010 in the records of Albacete, Murcia, and Navarra, up to 2012–2014 in the case of Tarragona,^25^ and from 2001 to 2015 for the Basque Country.^26^ Estimates of cancer incidence in Spain in recent years are made from the incidence data of the provinces with population cancer registries.

##### 3.2.3.2 Urinary bladder tumor coding rules.

For the coding of tumors, the ICD‐O‐3 (ICD‐O‐3.1. since 2013) is also used. Initially, for the coding of urinary bladder tumors, the recommendations of the 1995 ENCR were followed: all bladder tumors should be recorded regardless of histological type and level of infiltration (Table 4).^27^ Tumors /1, /2, /3 were coded according to the pathological description and the level of infiltration. A particular case was carcinoma in situ, which was coded as 8010/2. The coding of urothelial tumors in the different Spanish REDECAN registries is quite homogeneous, coding infiltrates as /3. Problematic cases appear when the degree of infiltration is not specified, the degree is not specified, or neither degree nor infiltration is specified.^28^ Thus, for example, if a urothelial tumor is grade I or grade II, or the grade is unknown, and information on infiltration is also lacking, in the Navarra registry marked this case with an asterisk (*), the Canary Islands code it as /1 and Murcia as /3. In contrast, a grade III urothelial tumor for which there is no information on infiltration, will be coded as /3 in the Navarra and Murcia registries, but as /1 in the Canary Islands. Based on all these classifications, in 2012 REDECAN proposed a consensus regarding the coding of noninfiltrating urothelial tumors (Table 5).^28^

**TABLE 4:** Recommendations for coding bladder tumors of European Network of Cancer Registries (ENCR) (1995)^27^

All bladder tumors should be registered, whatever the histological type and level of invasion.
**Principles:** The coding of tumor behavior (/1, /2, /3) takes into account both the anatomopathological definition and the extent of invasion. It is, therefore, essential to have access to reports of any pathological examinations
**Rules:** **Tumor behavior code: /1** Normal or slightly abnormal histology: low grade papillary urothelial tumors, not invasive. In the various anatomopathological classifications, these tumors are called: . benign or simple papillomas, . papillary urothelial tumors, . stage I carcinoma (BRODERS’ classification), . well‐differentiated papillary carcinoma (JEWETT's classification), . grade I carcinoma (in the WHO classification), or . classes I and IIs (CHOME's classification). Extent of invasion ‐ none**.** **Tumor behavior code: /2** Presence of mitoses and more markedly atypical cells than in the previous categories. It includes both high grade papillary urothelial tumors and flat tumors. Extent of invasion ‐ none**.** **Tumor behavior code: /3** Invasion present, whatever the anatomopathological definition.
**Particular cases:** **‐ Carcinoma in situ: /2** The particular entity which consists of carcinoma in situ displaying clear anaplasia of the superficial epithelium without the formation of a papillary structure and without invasion is coded to 8010/2. ‐ **Anatomopathological examination indicates the existence of a tumor, but it is not possible to determine the degree of malignancy on the specimen examined:** Code: /1 tumor benign or of uncertain malignancy **‐ Anatomopathological proof unavailable, but the clinical appearance is confirmed by the clinician:** 8000/0: No microscopically confirmation: tumor clinically benign. 8000/1: No microscopically confirmation: tumor clinically of uncertain behavior. 8000/3: No microscopically confirmation: tumor clinically malignant.

**TABLE 5:** Consensus on the coding of urothelial tumors proposed by REDECAN in 2012^28^

	ENCR	CIE‐O‐3	WHO	Grade	CONSENSUS
Urothelial papilloma Papilloma transitional cell, NOS (C67._)	8120/1 8130/1	8120/1	8120/0	(−)	8120/0 ‐
Papillary transitional cell neoplasm of low malignant potential	(−)	8130/1	8130/1	(−)	8130/1 ‐
Papillary transitional cell carcinoma, noninvasive (Grade I; OMS 1973) (pTa/0a)	8120/1 8130/1	8130/2 Non‐grade	8130/2	1	8130/2 1
Papillary transitional cell carcinoma of high grade*, noninvasive (Grade II/III; OMS 1973) (pTa/0a)	8120/2 8130/2	8130/2 Non‐grade	8130/2	3	8130/2 3
Carcinoma in situ Urothelial carcinoma in situ (pTis/0is)	8010/2	8010/2	8010/2	(−)	8010/2 ‐
Tumor noninvasive with degree of malignancy nondeterminate	‐−−/1	(−)	(−)	(−)	8010/1 ‐
Tumor (urothelial) with level of infiltration non‐determinate					8010/1 1 Low grade 8010/1 3 High grade
Tumor with degree of malignancy and level of infiltration non‐determinate					8010/1 ‐

Abbreviations: ENCR, European Network of Cancer Registries; ICD‐O‐3, International Classification of Diseases for Oncology, third edition; WHO, World Health Organization.

##### 3.2.3.3 Incidence of urinary bladder cancer

The estimates of cancer incidence in Spain for 2018 published by REDECAN in 2019^29^ are based on their own registries of 10 types of cancer. They are compared with the incidences of other countries obtained from ECIS. However, for the BC, due to the differences in the definition and in the inclusion criteria and in order to make the data comparable, the ECIS estimate for Spain has also been used. Thus, the estimated incidence rate of BC by the ECIS for 2018 is 70.2 for men and 12.5 for women. In contrast, the value of the REDECAN estimate is 91.6 for men and 15.8 for women.

Reviewing the BC incidence data provided by CI5 in volumes X^14^ and XI^10^ for the different registries that are part of REDECAN and the data provided by REDECAN in different publications,^25^, ^26^, ^29^, ^30^, ^31^ there may be differences in the incidences provided depending on the tumors included. In the BC incidence registries of Ciudad Real, Cuenca, Granada, and Tarragona presented in volume X of IC5,^14^ neoplasms of uncertain or unknown behavior are not included, but they are in the registries of volume XI. In the Canary Islands, the incidences of BC reported in volumes X and XI of the IC59^14^—where neoplasms of uncertain or unknown behavior are included together with invasive tumors—are higher than those that appear in their respective regional registries and in the 2018 estimates^30^, ^31^(Table 3).

## 4 DISCUSSION

BC has its own characteristics that differentiate it from other types of cancer, especially due to the difficulty involved in defining malignancy. Unlike other types of cancer, flat carcinomas in situ (/2) and some uncertain tumors (/1), although not coded as invasive (/3), may behave clinically as malignant. In addition, not only BC has a high rate of recurrence, but also generally presents as multicentric, and in some cases with a strong potential for infiltration, even if the invasion of the submucosa is not observed in the anatomopathological sample. We have recently carried out a study on the high incidence of BC in an industrialized Mediterranean area, which has led us to consider the comparability of the incidence of BC between the different registries.^32^

The most limiting factor for interpreting BC incidence rates and estimates is undoubtedly the coding of noninvasive tumors (considering the level of invasion and grade recorded), which determines their inclusion or not in the registries as “bladder cancer”. Superficial neoplasms of the bladder were initially excluded from cancer registries. Afterward, and probably linked to a better understanding of the behavior of noninvasive papillary tumors and carcinoma in situ, changes were made in the classification of tumor behavior and, therefore, the registration practices related to the coding of the invasiveness of the BC were modified. Thus, in IARC world registries published in CI5, the inclusion or not of superficial tumors was modified. But since no distinction is made between non‐muscle‐invasive and muscle‐invasive carcinomas of the bladder, separate epidemiology of each type cannot be obtained in a systematic way. Therefore, estimates of BC incidence are very difficult to interpret without comprehensive information on how superficial or uncertain bladder tumors have been treated by each of the registries. In general, changes in registry procedures are more likely to affect comparisons between registries than trends in a single registry over time, provided practices have not changed over time.^10^

Regarding the coding rules for multiple tumors, those used by SEER^22^ in cancer registries in the United States are different from those of IARC^5^ that were used in the rest of the world, which influenced their incidence rates. The SEER rules result in somewhat higher incidence rates not only because they diagnose multiple tumors if the patient has one micropapillary urothelial carcinoma and one bladder urothelial carcinoma, but because they allow multiple incident cancers to occur in the same body location, as long as the new case occurs 2 months to 3 years after a previous diagnosis, whereas IARC rules allow only one cancer per site during a patient's lifetime, unless there are several cancers of different histological types.

Recently, the ENCR has published new recommendations for unifying the great variability among registries in the criteria for recording and reporting urinary tract tumors.^33^ They insist on the importance of differentiating between recording (registration, coding, and classification) and reporting (counting in the statistics of incidence and survival) tumors. A cancer registry can record several tumors of the urothelium (of different site, grade, or invasion) of the same patient but according to international criteria and for the purposes of comparability, only one or a part of them is reported. These recommendations must be applied to all BC tumors with an incidence date on or after the January 1, 2022.^33^

Finally, another limiting factor in registry comparability, common to any type of cancer, is that most registries, except SEER, are affected by reporting delays. The SEER releases its cancer data with a delay of up to 28 months while the latest IARC actual incidence data is from 2010 to 2012. More recent data correspond to estimates of incidence based on previous data, which makes interpretation difficult.

Tobacco smoking is the most well‐established risk factor for BC, causing 50–65% of male cases and 20–30% of female cases.^34^ Occupational exposure is the second most important risk factor for BC and it is likely to occur in occupations in which dyes, rubbers and textiles, paints, leathers, and chemicals are used.^34^ Any of these registries consider these confounding factors in relation to BC incidence. However, these international or national associations were created for the development of cancer registration and its application to studies of well‐defined populations. They conduct programs of research concentrating particularly on the epidemiology of cancer and the study of potential carcinogens in the human environment with many publications about the different risk factors implicated in the incidence of BC.

Finally, in addition to the different BC coding rules, there are other factors that influence the heterogeneity of BC incidences. There are also problems in the comparability of the pathological anatomy of BC. On one hand, there are multiple anatomical pathological classifications to use. On the other hand, there may be different degrees of pathological differentiation within the same tumor and, moreover, bladder tumors are frequently multicentric. When examining it, there may be difficulties in assessing the degree of infiltration due to the probable deterioration of the biopsy material and/or its size or depth. Lastly, not all pathological reports, coding in pathological anatomy services and extraction of registry data will have the same quality.

In conclusion, the BC registry, unlike other types of cancer, is complex due to the peculiarities of urothelial tumors that make it difficult to define their malignancy. The methodological differences used by the different registries regarding the inclusion of papillary tumors, carcinomas in situ and indeterminate, complicate the comparisons of the incidence of BC between registries. This limitation is worsened by the fact that urothelial tumors are frequently multicentric and have a high rate of recurrence, but the rules for defining single or multiple tumors are not homogeneous among the different cancer registries. Changes in BC coding and recording rules over time interfere with incidence estimates. Finally, we think that cancer registries are increasingly recognized as a tool that will help clinicians assess individualized survival predictions. Furthermore, a good cancer registry could provide better surveillance strategies for BC patients.

## AUTHOR CONTRIBUTIONS

**Jose Maria Caballero:** Conceptualization (lead); data curation (lead); investigation (lead); methodology (lead); writing – original draft (lead); writing – review and editing (lead). **Jose Maria Gili:** Writing – original draft (supporting); writing – review and editing (supporting). **Juan Camilo Pereira:** Writing – review and editing (supporting). **Alba Gomáriz:** Writing – review and editing (supporting). **Carlos Castillo:** Writing – review and editing (supporting). **Montserrat Martin‐Baranera:** Conceptualization (lead); data curation (lead); investigation (lead); methodology (lead); writing – original draft (lead); writing – review and editing (lead).

## FUNDING INFORMATION

The authors report no funding.

## CONFLICT OF INTEREST

The authors declare that they have no conflict of interest.

## ETHICS STATEMENT

The manuscript received the Ethical Approval from the Ethics and Research Committee of the Fundació Assitencial Mútua de Terrassa (07/2018).

## Data Availability

Data sharing is not applicable to this article as no new data were created or analyzed in this study.

## References

[1] Parkin DM . The role of cancer registries. Int J Clin Oncol. 2008;13:102‐111. 18463952 10.1007/s10147-008-0762-6

[2] Navarro C , Martos C , Ardanaz E , et al. For the Spanish cancer registries working group. Population‐based cancer registries in Spain and their role in cancer control. Ann Oncol. 2010;21(Suppl 3):iii3‐iii13. doi:10.1093/annonc/mdq094 20427357

[3] Leal YA , Fernández‐Garrote LM , Mohar‐Betancourt A , Meneses‐García A . The importance of registries in cancer control. Salud Publica Mex. 2016;58:309‐316. 27557391 10.21149/spm.v58i2.7802

[4] Brawley OW . The cancer registry as a cancer‐control tool. Cancer. 2016;122(9):1343‐1345. doi:10.1002/cncr.29968 26959554

[5] Zanetti R , Sacchetto L , Coebergh JW , Rosso S . To accelerate cancer prevention in Europe: challenges for cancer registries. Eur J Cancer. 2018;104:151‐159. 30352383 10.1016/j.ejca.2018.09.001

[6] World Health Organization. International Association of Cancer Registries. European Network of Cancer Registries . International rules for multiple primary cancer (ICD‐O third edition) International Agency for Research on Cancer.IARC; 2004.

[7] Crow P , Ritchie AWS . National and international variation in the registration of bladder cancer. BJU Int. 2003;92:563‐566. 14511034 10.1046/j.1464-410x.2003.04421.x

[8] Chavan S , Bray F , Lortet‐Tieulent JL , Goodman M , Jemal A . International variations in bladder cancer incidence and mortality. Eur Urol. 2014;66:59‐73. 24451595 10.1016/j.eururo.2013.10.001

[9] PRISMA [Internet]. [Accessed 17 September 2021]. Available from: http://prisma‐statement.org/prismastatement/Checklist.aspx

[10] Bray F , Colombet M , Mery L , et al. editors. Cancer Incidence in Five Continents. Vol. XI. IARC Scientific Publication No. 166. International Agency for Research on Cancer; 2021. Available from: https://publications.iarc.fr/597 [Accessed 31 January 2021].

[11] Ferlay J , Ervik M , Lam F , et al. Global Cancer Observatory: Cancer Today. International Agency for Research on Cancer; 2020. Available from: https://gco.iarc.fr/today [Accessed 31 January 2021].

[12] Parkin DM , Whelan SL , Ferlay J , et al. Cancer Incidence in Five Continents. Vol. VIII. IARC Scientific Publication No. 155. International Agency for Research on Cancer. 2002. Available from: https://publications.iarc.fr/Book‐And‐Report‐Series/Iarc‐Scientific‐Publications/Cancer‐Incidence‐In‐Five‐Continents‐Volume‐VIII‐2002 [Accessed 20 December 2020].

[13] Curado MP , Edwards B , Shin HR , et al. Cancer Incidence in Five Continents. Vol. IX. IARC Scientific Publication No. 160. International Agency for Research on Cancer. 2007. Available from: https://www.iarc.who.int/cards_page/iarc‐publications/ [Accessed 20 December 2020].

[14] Forman D , Bray F , Brewster DH , et al. editors. Cancer Incidence in Five Continents. Vol. X. IARC Scientific Publication No. 164. International Agency for Research on Cancer. 2014. Available from: https://ci5.iarc.fr/CI5I‐X/old/vol10/CI5vol10.pdf [Accessed 20 December 2020].

[15] World Health Organization . International Statistical Classification of Diseases and Related Health Problems. WHO; 1992.

[16] International Statistical Classification of Diseases and Related Health Problems. 10th Revision. 10 February 2010. Available from: https://icd.who.int/browse10/2010/en [Accessed 13 November 2020].

[17] Fritz A , Percy C , Jack A , et al. International Classification of Diseases for Oncology. Third ed. World Health Organization; 2013.

[18] Muir CS , Waterhouse J , Mack T , et al. eds. Cancer Incidence in Five Continents. Vol. V. IARC Scientific Publications. No. 88. International Agency for Research on cancer; 1987. Available from: https://ci5.iarc.fr/CI5I‐X/Default.aspx [Accessed 20 December 2020].

[19] Parkin DM , Whelan SL , Ferlay J , et al. Cancer Incidence in Five Continents. Vol. VII IARC Scientific Publications. No. 143. International Agency for Research on Cancer. Research on cancer; 1997.

[20] Islam F , ackn E , Sung H , et al. Annual report to the nation on the status of cancer. Part 1: National Cancer Statistics. J Natl Cancer Inst. 2021;113:1648‐1669. doi:10.1093/jnci/djab131 34240195 PMC8634503

[21] Ruhl JL , Callaghan C , Hurlbut A , et al. eds. Summary Stage 2018: Codes and Coding Instructions. National Cancer Institute; 2020. Available from: https://seer.cancer.gov/ [Accessed 15 December 2020].

[22] Johnson CH , Peace S , Adamo P , et al. The 2007 Multiple Primary and Histology Coding Rules. National Cancer Institute. Surveillance. Epidemiology and End Results Program; 2007.

[23] Howlader N , Noone AM , Krapcho M , et al. SEER Cancer Statistics Review. National Cancer Institute; 1975‐2018.

[24] ECIS‐European Cancer Information System . Available from: https://ecis.jrc.ec.europa.eu [Accessed 15 December 2020].

[25] Galceran J , Carulla M , Bigorra J , et al. El càncer a Tarragona 2012‐2014 i les seves projeccions. Servei d'Epidemiologia i Prevenció del Càncer. Hospital Universitari de Sant Joan de Reus. Reus; 2020.

[26] Lopez de Munain A , Audicana C . Cáncer en la Comunidad Autonoma de Euskadi 2001‐2017. Registro de Cáncer de Euskadi. Registro de Mortalidad de Euskadi. 2019. Departamento de Salud del Gobierno Vasco. Mayo. Available from: http://www.euskadi.eus/informacion/registries‐de‐cancer/web01‐a3regepi/es [Accessed 15 February 2021].

[27] Pheby D , Martinez C , Roumagnac M , Schouten L . ENCR Working Group. Recommendations for Coding Bladder Cancers. European Network of Cancer Registries (ENCR). 1995.

[28] Vilardell L , Franch P , Marcos‐Gragera R . Curso de codificación y registro de las neoplasias uroteliales. Talavera de la Reina 12–13 de Noviembre de 2012. Curso formación REDECAN. Available from: https://redecan.org/redecan.org/es/Clasificacion_y_codificacion_de_las_neoplasias_uroteliales71bd.pdf?file=743&area=212 [Accessed 15 December 2020].

[29] Estimaciones de la incidencia del cáncer en España . Red Española de Registries de Cáncer (REDECAN). 2019. Available from: https://redecan.org/redecan.org/es/index.html [Accessed 15 December 2020].

[30] Registro Poblacional de Cáncer de Canarias – Datos de incidencia. Available from: https://www3.gobiernodecanarias.org [Accessed 15 February 2021].

[31] Alemán Herrera A , Rojas Martín MD . Servicio de Epidemiología y Prevención. Dirección General de Salud Pública. Registro poblacional del cancer de Canarias. Estudio de incidencia del cáncer de Canarias 2018. 2019. Available from: https://www3.gobiernodecanarias.org [Accessed 15 February 2021].

[32] Caballero JM , Pérez‐Márquez M , Gili JM , et al. Environmental factors involved in the high incidence of bladder cancer in an industrialized area in north‐eastern Spain. J Environ Public Health. 2022;2022:1051046. 35844948 10.1155/2022/1051046 PMC9282998

[33] Eden M , Daubisse‐Marliac L , Galceran J , et al. ENRC recommendations: recording and reporting of urothelial tumours of the urinary tract Available from: https://www.encr.eu/sites/default/files/Recommendations/ENCR%20Recommendation_UT_Jun2022_EN.pdf [Accessed 19 July 2022]. 10.3389/fonc.2022.1046239 PMC9727225 36505871

[34] Witjes JA , Bruins HM , Cathomas R , et al. European Association of Urology guidelines on muscle‐invasive and metastatic bladder cancer: Summary of the 2020 guidelines. Eur Uro. 2021;79(1):82‐104. 10.1016/j.eururo.2020.03.055 32360052

